# Supplementary figures and images for: The transcription factor SKN-1 drives lysosomal enlargement during aging to maintain function
Source: PLoS Biol. 2025 Dec 5;23(12):e3003540. doi: 10.1371/journal.pbio.3003540 (PMC12694847; doi:10.1371/journal.pbio.3003540)

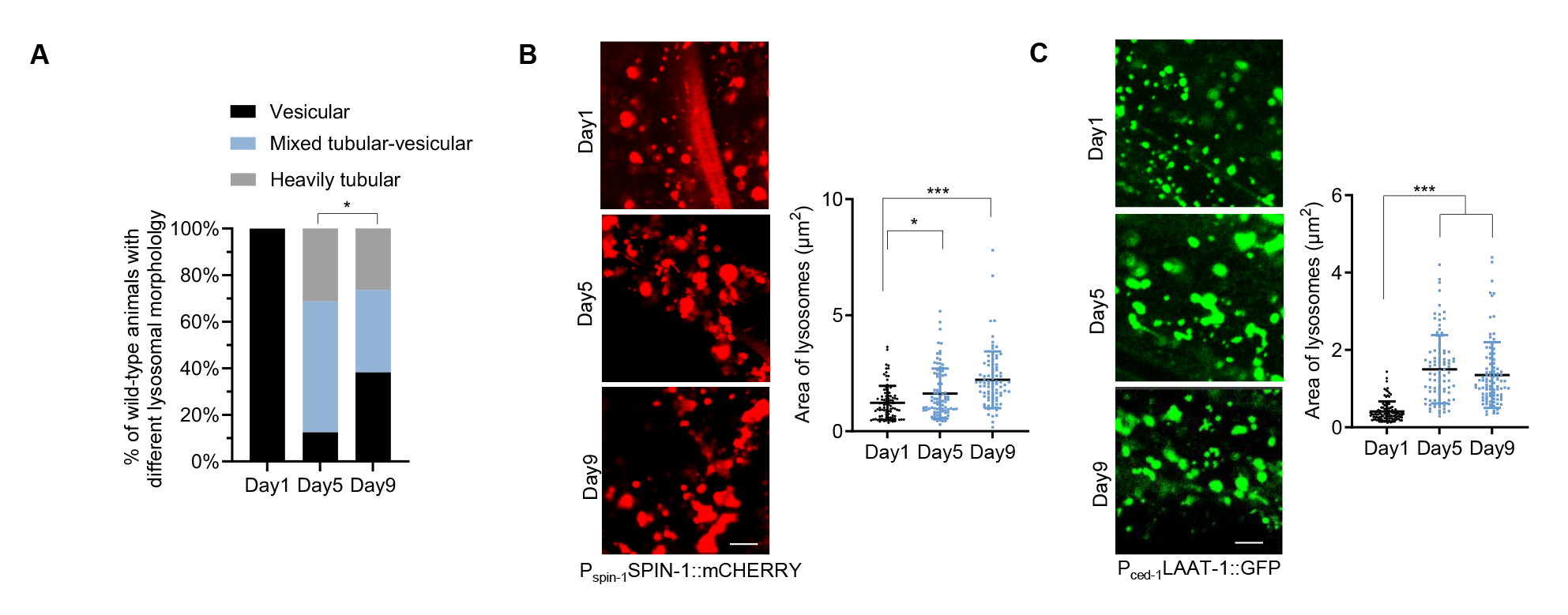

Supplement: S1 Fig — (A) Ratio of animals exhibiting different lysosomal morphologies in the intestine during aging. Chi-square and Fisher’s exact test. n = 28–34 animals. (B) The morphology and size of vesicular lysosomes in the intestine during aging. Left: representative images. Right: quantification data. One-way ANOVA analysis followed by Dunnett’s multiple comparisons post hoc test. n = 84–88 lysosomes. (C) The morphology and size of vesicular lysosomes in the muscle during aging. Left: representative images. Right: quantification data. One-way ANOVA analysis followed by Dunnett’s multiple comparisons post hoc test. n = 90–102 lysosomes. Data are presented as mean ± SD. *p < 0.05, ***p < 0.001. Scale bar = 5 μm for panels (B) and (C). The numerical data presented in this figure can be found in S1 Data. (TIF) [file pbio.3003540.s001.tif]

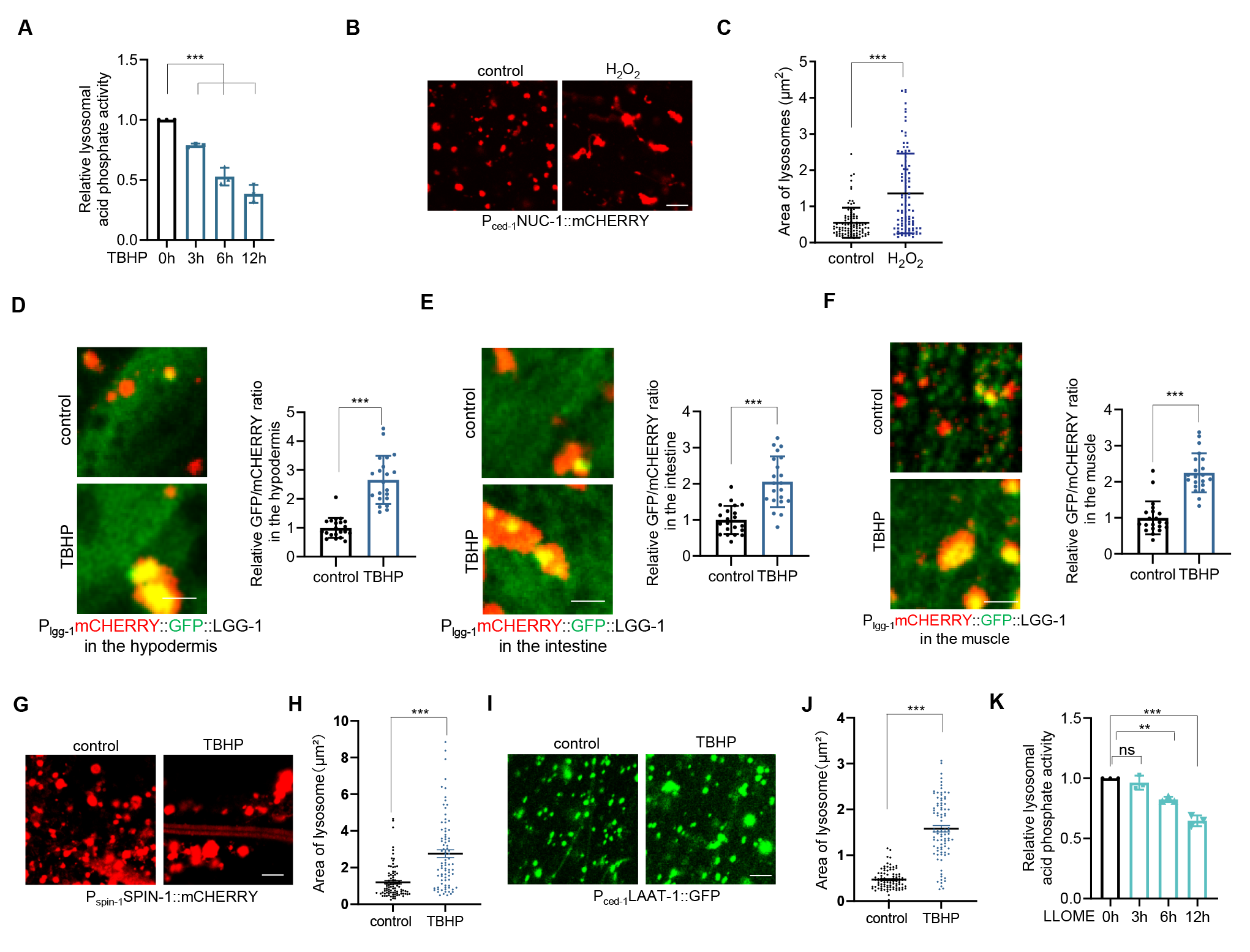

Supplement: S2 Fig — (A) Effect of chronic TBHP treatment on the activity of lysosomal acid phosphate in day 1 adults. One-way ANOVA analysis followed by Dunnett’s multiple comparisons post hoc test. n = three independent experiments. (B and C) Effect of 6-hour hydrogen peroxide treatment on lysosomal morphology (B) and size (C) in the hypodermis of day 1 adults. Unpaired t test analysis. n = 100 lysosomes. (D–F) Effect of 6-hour TBHP treatment on the GFP/mCHERRY ratio in the hypodermis (D), intestine (E), and muscle (F) of mCHERRY::GFP::LGG-1 day 1 adults. Left: representative images. Right: quantification data. Unpaired t test analysis. n = 21 animals. (G–J) Effect of 6-hour TBHP treatment on lysosomal morphology and size in the intestine (G, H) and muscle (I, J) of day 1 adults. (G, I): representative images. (H, J): quantification data. Unpaired t test analysis. n = 83–91 lysosomes for (H) and 90 lysosomes for (J). (K) Effect of LLOME treatment on the activity of lysosomal acid phosphate in day 1 adults. One-way ANOVA analysis followed by Dunnett’s multiple comparisons post hoc test. Data are presented as mean ± SD. **p < 0.01, ***p < 0.001. Scale bar = 5 μm for panels (B), (G), and (I); 1.25 μm for panels (D–F). The numerical data presented in this figure can be found in S1 Data. (TIF) [file pbio.3003540.s002.tif]

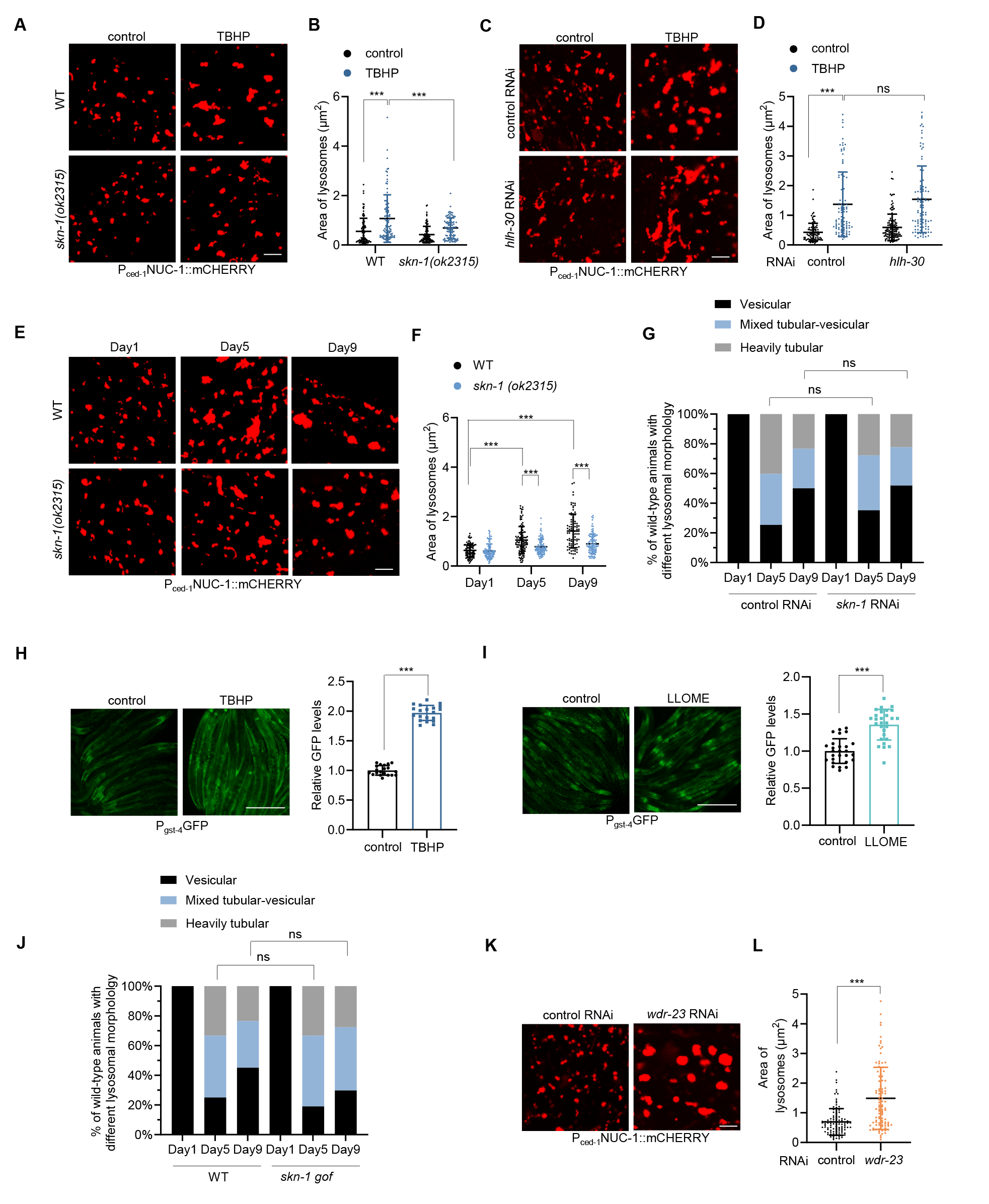

Supplement: S3 Fig — (A and B) Effect of skn-1(ok2315) mutation on lysosomal morphology (A) and size (B) in response to TBHP treatment in the hypodermis of day 1 adults. Two-way ANOVA analysis followed by Tukey’s multiple comparisons post hoc test. n = 93−99 lysosomes. (C and D) Effect of hlh-30 RNAi on lysosomal morphology (C) and size (D) in response to 6-hour TBHP treatment in the hypodermis of day 1 adults. Two-way ANOVA analysis followed by Tukey’s multiple comparisons post hoc test. n = 100−132 lysosomes. (E and F) Effect of skn-1(ok2315) mutation on vesicular lysosomal morphology (E) and size (F) in the hypodermis during aging. Two-way ANOVA analysis followed by Tukey’s multiple comparisons post hoc test. n = 90−104 lysosomes. (G) Effect of skn-1 RNAi on the ratio of animals exhibiting different lysosomal morphologies in the hypodermis during aging. Chi-square and Fisher’s exact test. n = 54−67 animals. (H) 6-hour TBHP treatment induces expression of gst-4p::GFP in day 1 adults. Left: representative images. Right: quantification of fluorescent intensity. Unpaired t test analysis. n = 20 animals. (I) 6-hour LLOME treatment induces expression of gst-4p::GFP in day 1 adults. Left: representative images. Right: quantification of fluorescent intensity. Unpaired t test analysis. n = 27−28 animals. (J) Effect of skn-1 gof mutation on the ratio of animals exhibiting different lysosomal morphologies in the hypodermis during aging. Chi-square and Fisher’s exact test. n = 51−52 animals. (K and L) Effect of wdr-23 RNAi on lysosomal morphology (K) and size (L) in the hypodermis of day 1 adults. Unpaired t test analysis. n = 100 lysosomes. Data are presented as mean ± SD. ***p < 0.001. Scale bar = 5 μm for panels (A), (C), (E), and (K); 150 μm for panels (H) and (I). The numerical data presented in this figure can be found in S1 Data. (TIF) [file pbio.3003540.s003.tif]

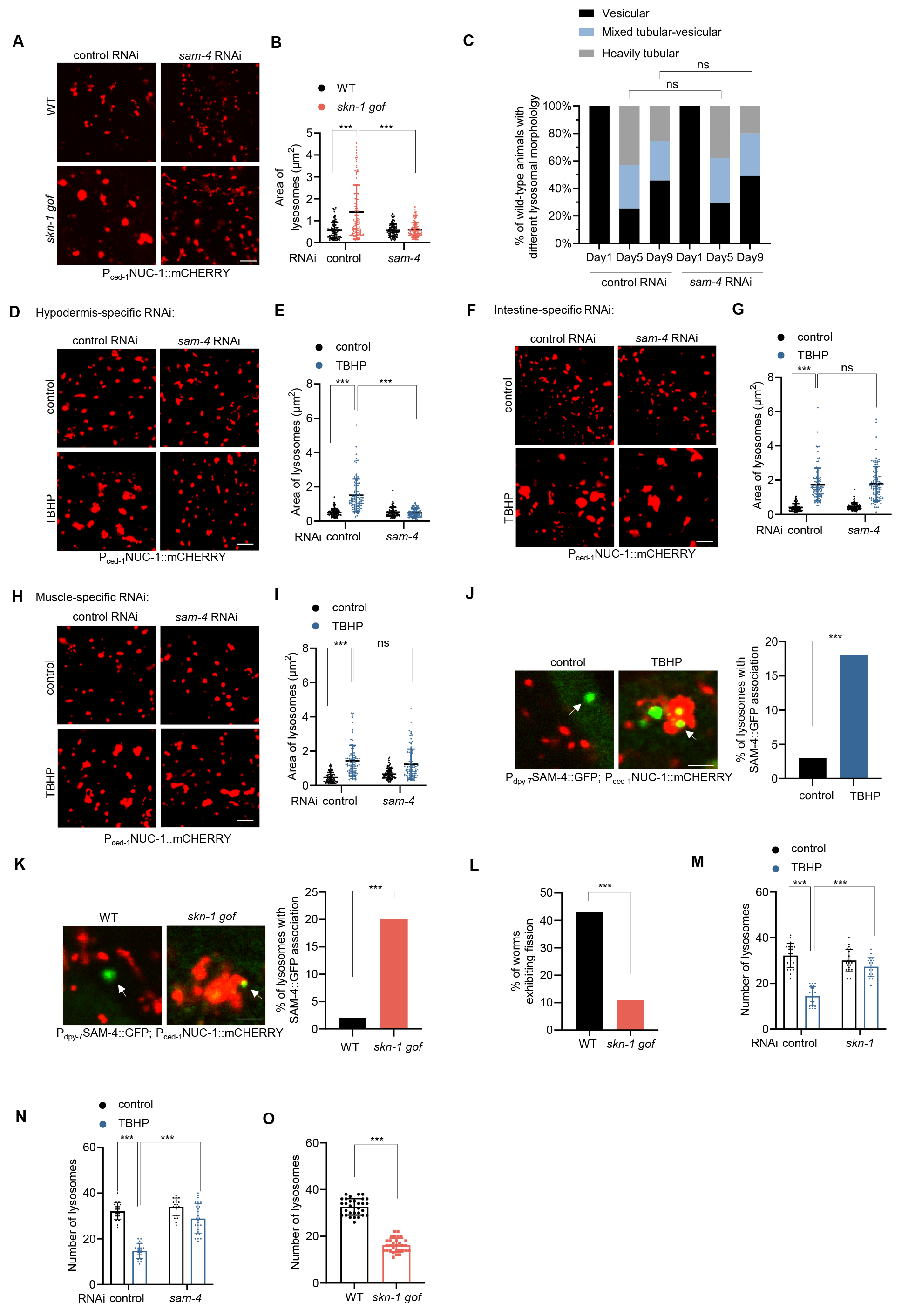

Supplement: S4 Fig — (A and B) Effect of sam-4 RNAi on lysosomal morphology (A) and size (B) in response to skn-1 gof mutation in the hypodermis of day 1 adults. Two-way ANOVA analysis followed by Tukey’s multiple comparisons post hoc test. n = 100 lysosomes. (C) Effect of sam-4 RNAi on the ratio of animals exhibiting different lysosomal morphologies in the hypodermis during aging. Chi-square and Fisher’s exact test. n = 55–63 animals. (D–I) Effects of hypodermis-specific (D, E), intestine-specific (F, G), and muscle-specific (H, I) sam-4 RNAi on hypodermal lysosomal morphology and size in response to 6-hour TBHP treatment in day 1 adults. (D, F, and H): representative images. (E, G, and I): quantification data. Two-way ANOVA analysis followed by Tukey’s multiple comparisons post hoc test. n = 91–97 lysosomes for (E), 89–93 lysosomes for (G), and 84–93 lysosomes for (I). (J) Effect of 6-hour TBHP treatment on lysosomal association with SAM-4::GFP in the hypodermis of day 1 adults. Left: representative images. White arrows indicate SAM-4::GFP signals. Right: quantification of lysosomal association with SAM-4::GFP. Chi-square and Fisher’s exact test. n = 100 lysosomes. (K) Effect of skn-1 gof mutation on lysosomal association with SAM-4::GFP in the hypodermis of day 1 adults. Left: representative images. White arrows indicate SAM-4::GFP signals. Right: quantification of lysosomal association with SAM-4::GFP. Chi-square and Fisher’s exact test. n = 100 lysosomes. (L) Effect of skn-1 gof mutation on lysosomal fission in the hypodermis of day 1 adults. Chi-square and Fisher’s exact test. n = 19–28 animals. (M and N) Effects of skn-1 (M) and sam-4 (N) RNAi on lysosomal number in response to 6-hour TBHP treatment in the hypodermis of day 1 adults. Two-way ANOVA analysis followed by Tukey’s multiple comparisons post hoc test. n = 26–35 animals for (M), and 23–30 animals for (N). (O) Effect of skn-1 gof mutation on lysosomal number in the hypodermis of day 1 adults. Unpaired t test analysis. n = [file pbio.3003540.s004.tif]

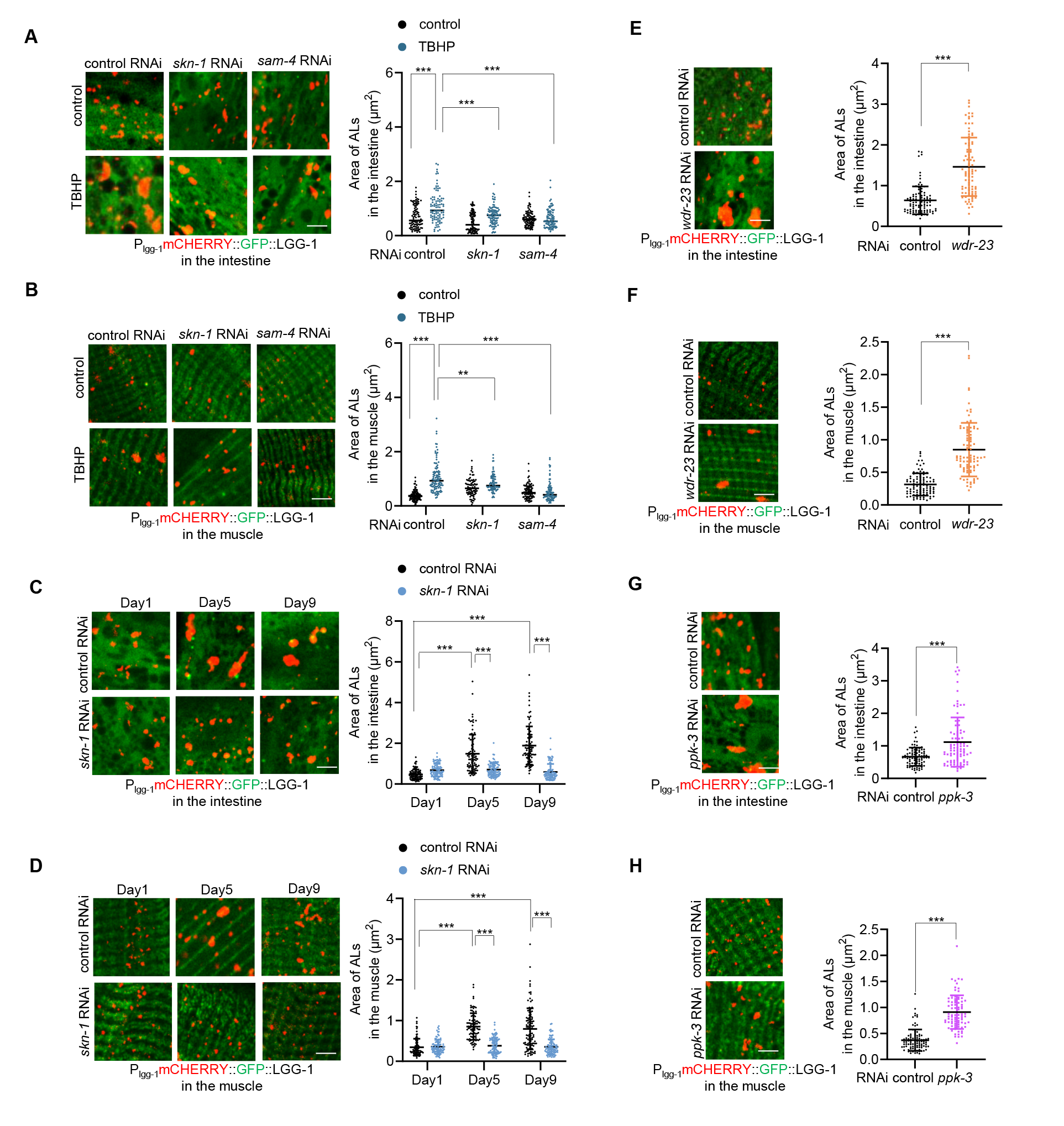

Supplement: S5 Fig — (A and B) Effects of skn-1 and sam-4 RNAi on AL size in the intestine (A) and muscle (B) of day 1 adults in response to 6-hour TBHP treatment. Left: representative images. Right: quantification data. Two-way ANOVA analysis followed by Tukey’s multiple comparisons post hoc test. n = 93–98 lysosomes for (A) and 79–110 lysosomes for (B). (C and D) Effect of skn-1 RNAi on AL size in the intestine (C) and muscle (D) during aging. Left: representative images. Right: quantification data. Two-way ANOVA analysis followed by Tukey’s multiple comparisons post hoc test. n = 89–93 lysosomes for (C) and 92–106 lysosomes for (D). (E and F) Effect of wdr-23 RNAi on AL size in the intestine (E) and muscle (F) of day 1 adults. Left: representative images. Right: quantification data. Unpaired t test analysis. n = 90–91 lysosomes for (E) and 89–95 lysosomes for (F). (G and H) Effect of ppk-3 RNAi on AL size in the intestine (G) and muscle (H) of day 1 adults. Left: representative images. Right: quantification data. Unpaired t test analysis. n = 85–89 lysosomes for (G) and 83–93 lysosomes for (H). Data are presented as mean ± SD. **p < 0.01, ***p < 0.001. Scale bar = 2.5 μm for all panels. The numerical data presented in this figure can be found in S1 Data. (TIF) [file pbio.3003540.s005.tif]

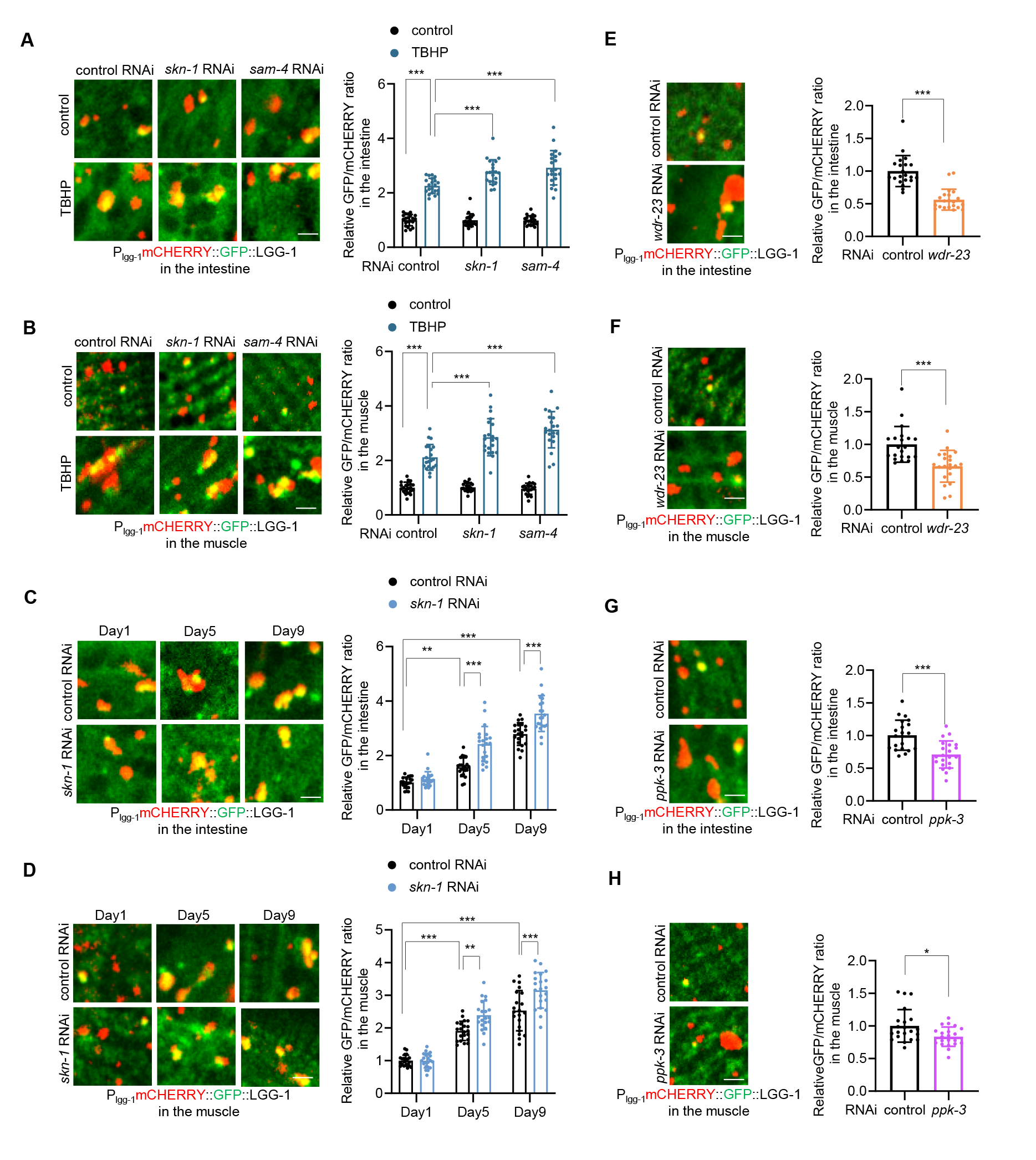

Supplement: S6 Fig — (A and B) Effects of skn-1 and sam-4 RNAi on the GFP/mCHERRY ratio in intestine (A) and muscle (B) of mCHERRY::GFP::LGG-1 day 1 adults in response to 6-hour TBHP treatment. Left: representative images. Right: quantification data. Two-way ANOVA analysis followed by Tukey’s multiple comparisons post hoc test. n = 21 animals. (C and D) Effect of skn-1 RNAi on the GFP/mCHERRY ratio in the intestine (C) and muscle (D) during aging. Left: representative images. Right: quantification data. Two-way ANOVA analysis followed by Tukey’s multiple comparisons post hoc test. n = 21 animals. (E-F) Effect of wdr-23 RNAi on the GFP/mCHERRY ratio in the intestine (E) and muscle (F) of day 1 adults. Left: representative images. Right: quantification data. Unpaired t test analysis. n = 21 animals. (G and H) Effect of ppk-3 RNAi on the GFP/mCHERRY ratio in the intestine (G) and muscle (H) of day 1 adults. Left: representative images. Right: quantification data. Unpaired t test analysis. n = 21 animals. Data are presented as mean ± SD. *p < 0.05, **p < 0.01, ***p < 0.001. Scale bar = 1.25 μm for all panels. The numerical data presented in this figure can be found in S1 Data. (TIF) [file pbio.3003540.s006.tif]

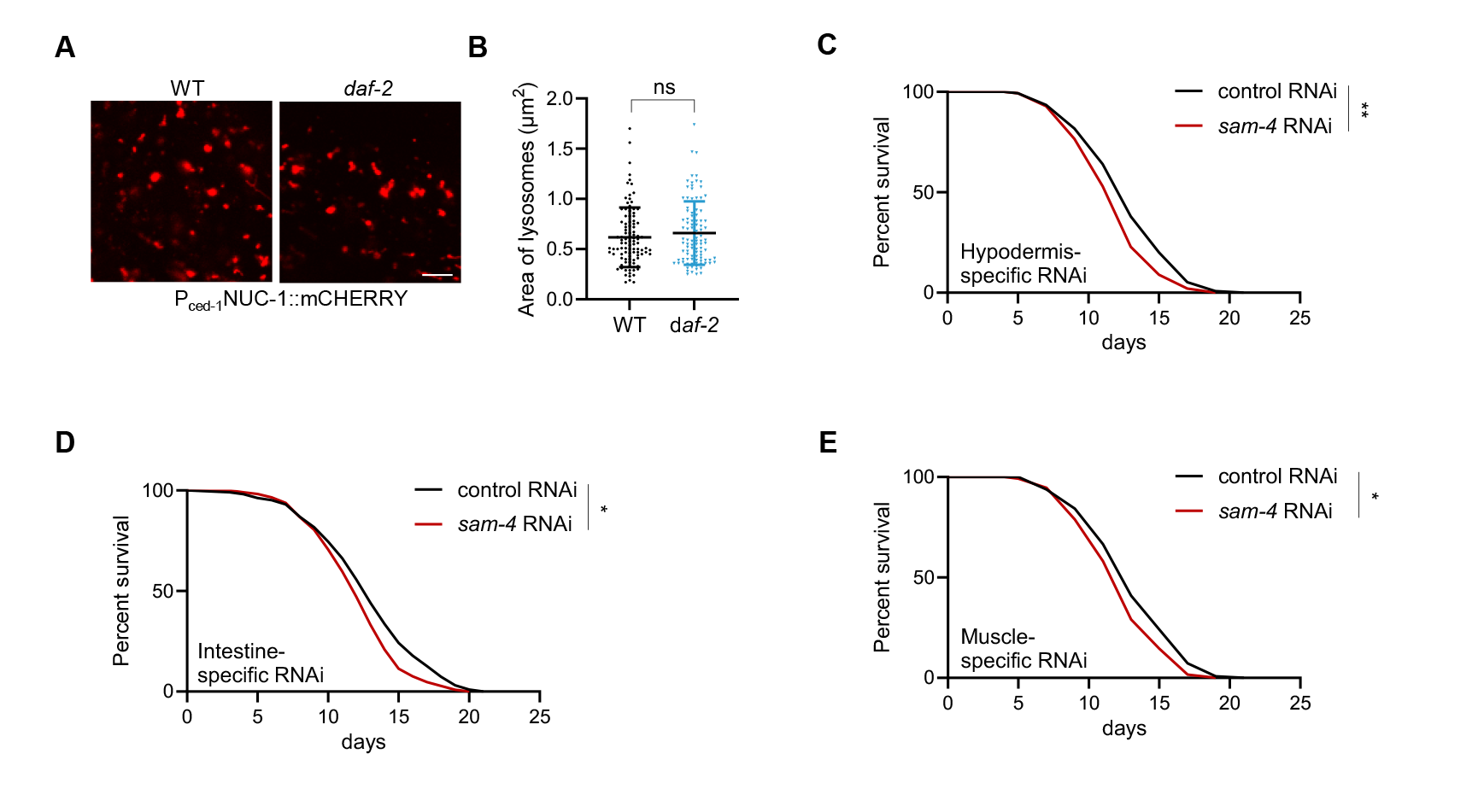

Supplement: S7 Fig — (A and B) Effect of daf-2 mutation on lysosomal morphology (A) and size (B) in the hypodermis of day 1 adults. Unpaired t test analysis. n = 100 lysosomes. (C–E) Effects of hypodermis-specific RNAi (C), intestine-specific RNAi (D) and muscle-specific RNAi (E) of sam-4 on the life span of WT animals. Log-rank (Mantel–Cox) test s followed by Bonferroni post hoc tests. Statistical analysis and additional repeats were listed in S1 Table. Data are presented as mean ± SD. *p < 0.05, **p < 0.01. Scale bar = 5 μm for panel (A). The numerical data presented in this figure can be found in S1 Data. (TIF) [file pbio.3003540.s007.tif]

**Figure2D**

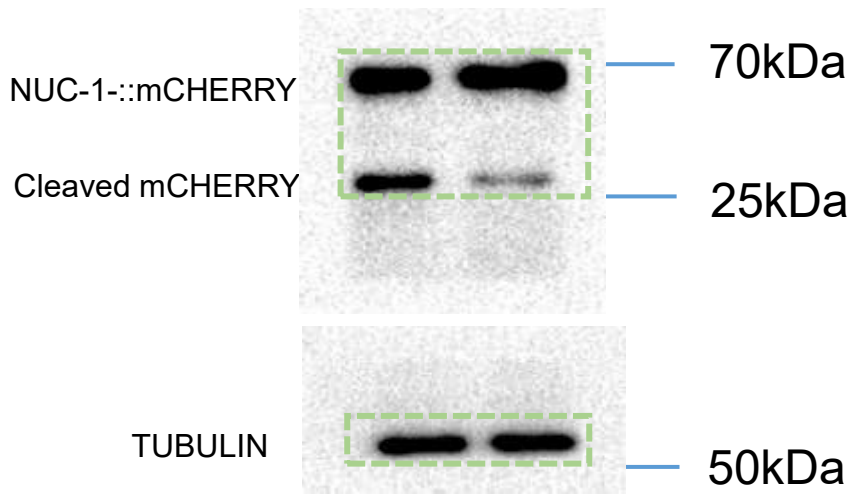

**Figure5E**

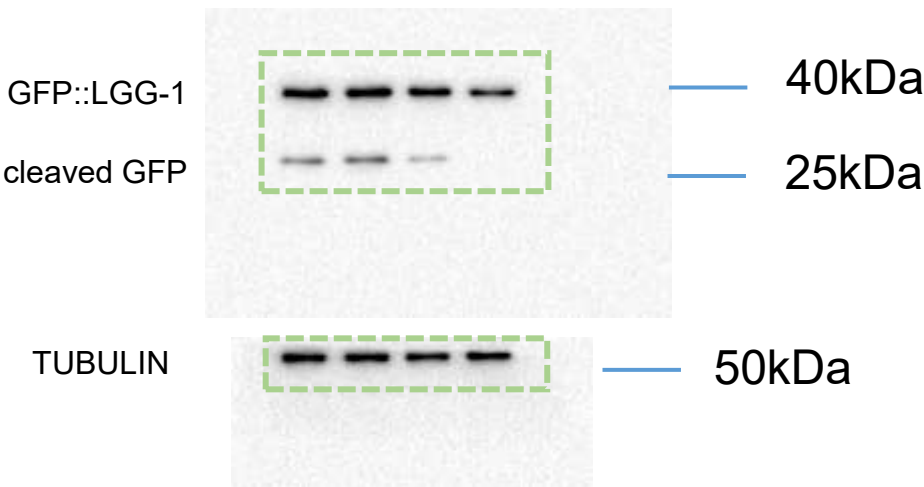

**Figure5F**

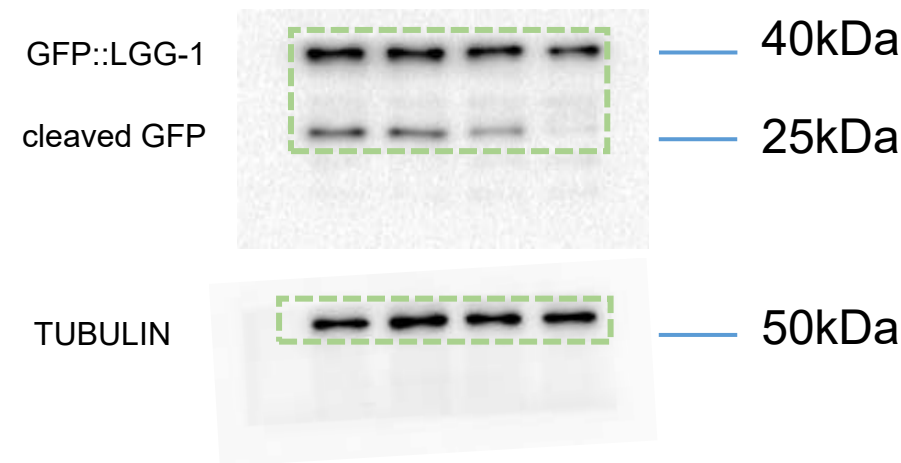

**Figure5G**

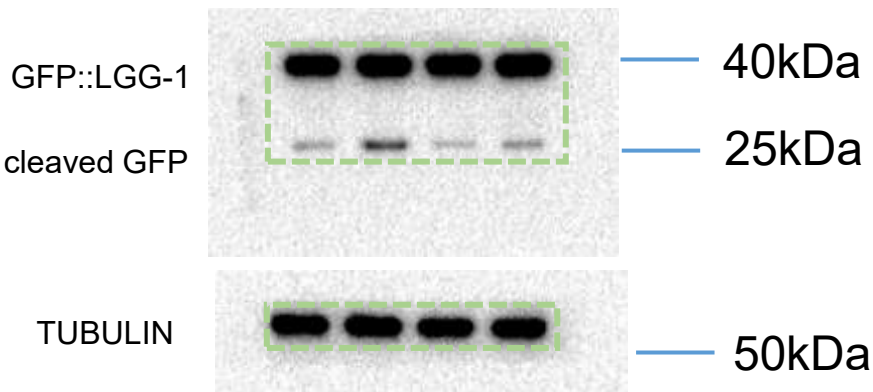

**Figure5H**

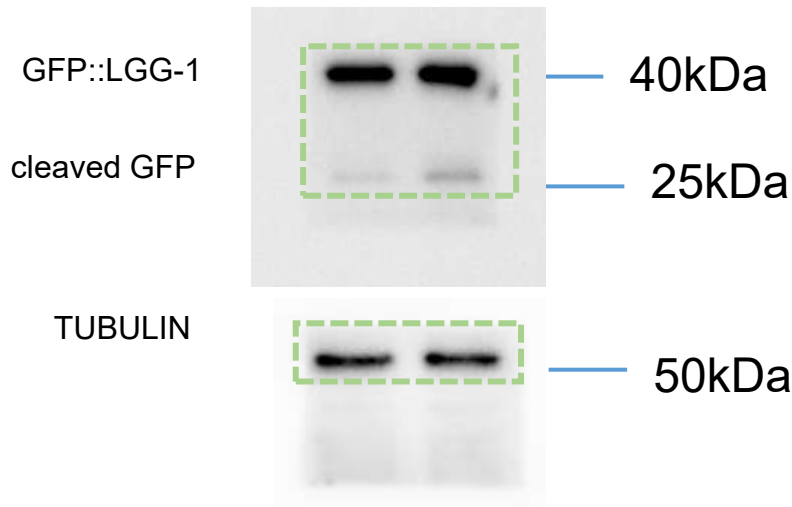

**Figure6H**

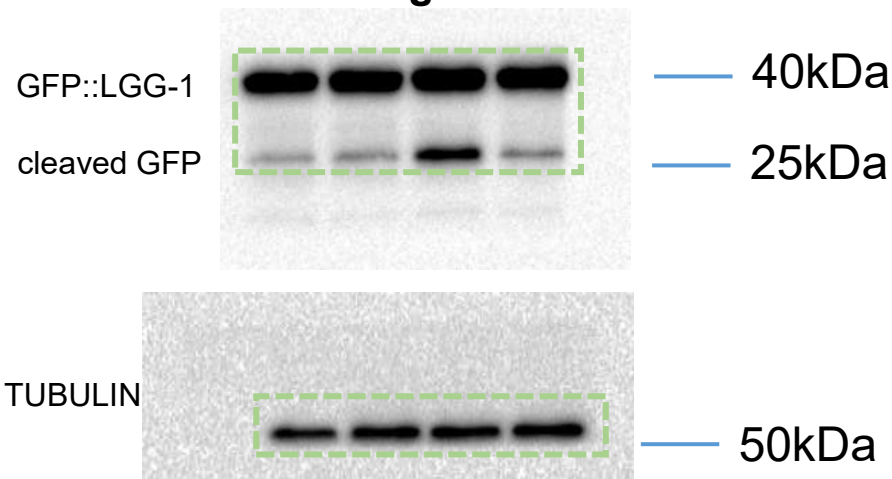

**Figure6I**

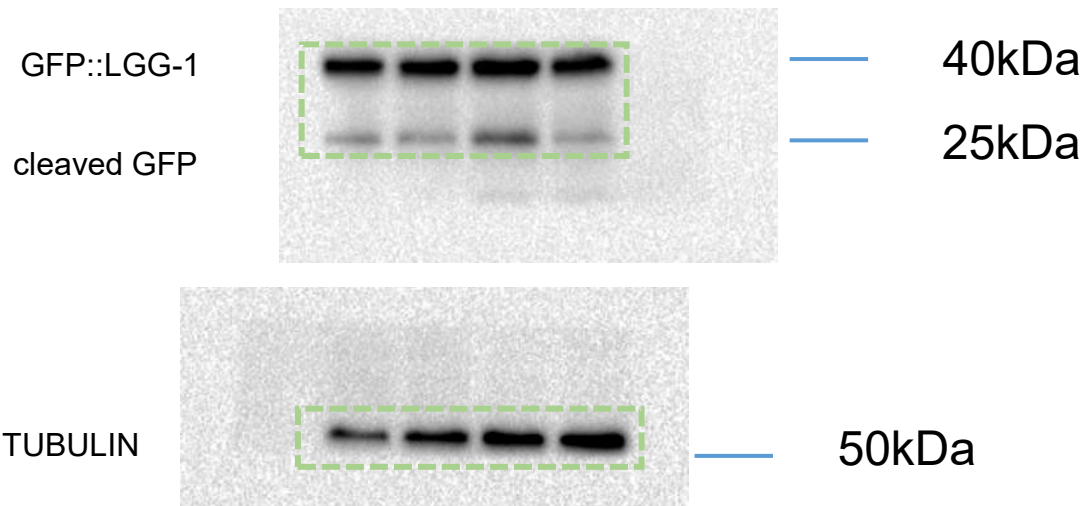

Supplement: S1 File — (PDF) [file pbio.3003540.s011.pdf]
