# Supplementary material for: The transcription factor SKN-1 drives lysosomal enlargement during aging to maintain function
Source: PLoS Biol. 2025 Dec 5;23(12):e3003540. doi: 10.1371/journal.pbio.3003540 (PMC12694847; doi:10.1371/journal.pbio.3003540)
Supplement: S1 Table — Repeats 1 were graphed in figures. (DOCX) [file pbio.3003540.s008.docx]

**S1 Table. Lifespan data. Repeats 1 were graphed in figures.**

| Figures | Strain/Treatment | Mean survival time  ± SEM (days) | # Worms  Censored/Total | Bonferroni P value |
| --- | --- | --- | --- | --- |
| 6J |  |  |  |  |
| Repeat 1 | control RNAi | 14.97 ± 0.44 | 4/112 |  |
|  | *sam-4* RNAi | 13 ± 0.34 | 6/129 | **<0.001^a^** |
| Repeat 2 | control RNAi | 14.99 ± 0.39 | 7/105 |  |
|  | *sam-4* RNAi | 13.37 ± 0.36 | 4/90 | **<0.01^a^** |
| Repeat 3 | control RNAi | 14.98 ± 0.45 | 0/120 |  |
|  | *sam-4* RNAi | 13.3 ± 0.34 | 1/143 | **<0.01^a^** |
| 6K |  |  |  |  |
| Repeat 1 | *glp-1* control RNAi | 24.26 ± 0.63 | 4/127 |  |
|  | *glp-1 sam-4* RNAi | 20.74 ± 0.52 | 3/118 | **<0.001^a^** |
| Repeat 2 | *glp-1* control RNAi | 24.11 ± 0.6 | 1/122 |  |
|  | *glp-1 sam-4* RNAi | 19.4 ± 0.42 | 3/123 | **<0.001^a^** |
| Repeat 3 | *glp-1* control RNAi | 24.02 ± 0.67 | 5/116 |  |
|  | *glp-1 sam-4* RNAi | 20.38 ± 0.61 | 9/110 | **<0.001^a^** |
| 6L |  |  |  |  |
| Repeat 1 | *daf-2* control RNAi | 26.28 ± 0.71 | 7/125 |  |
|  | *daf-2 sam-4* RNAi | 25.96 ± 0.74 | 5/139 | **0.864^a^** |
| Repeat 2 | *daf-2* control RNAi | 26.4 ± 0.67 | 6/132 |  |
|  | *daf-2 sam-4* RNAi | 25.94 ± 0.76 | 0/136 | **0.4322^a^** |
| Repeat 3 | *daf-2* control RNAi | 26.07 ± 0.7 | 12/138 |  |
|  | *daf-2 sam-4* RNAi | 24.75 ± 0.73 | 7/130 | **0.2305^a^** |
| S7C |  |  |  |  |
| Repeat 1 | control RNAi | 13.05 ± 0.27 | 13/148 |  |
|  | *sam-4* RNAi | 12.11 ± 0.23 | 11/160 | **<0.01^a^** |
| Repeat 2 | control RNAi | 13.25 ± 0.28 | 10/137 |  |
|  | *sam-4* RNAi | 12.24 ± 0.24 | 10/141 | **<0.01^a^** |
| Repeat 3 | control RNAi | 12.86 ± 0.29 | 15/133 |  |
|  | *sam-4* RNAi | 11.93 ± 0.27 | 9/119 | **<0.05^a^** |
| S7D |  |  |  |  |
| Repeat 1 | control RNAi | 12.91 ± 0.38 | 13/109 |  |
|  | *sam-4* RNAi | 12.16 ± 0.29 | 12/120 | **<0.05 ^a^** |
| Repeat 2 | control RNAi | 13.16 ± 0.39 | 12/105 |  |
|  | *sam-4* RNAi | 11.99 ± 0.26 | 9/123 | **<0.01^a^** |
| S7E |  |  |  |  |
| Repeat 1 | control RNAi | 13.35± 0.29 | 12/138 |  |
|  | *sam-4* RNAi | 12.54 ± 0.25 | 14/143 | **<0.05^a^** |
| Repeat 2 | control RNAi | 13.07 ± 0.29 | 8/132 |  |
|  | *sam-4* RNAi | 12.05 ± 0.25 | 9/133 | **<0.01^a^** |
| Repeat 3 | control RNAi | 13.16 ± 0.29 | 9/134 |  |
|  | *sam-4* RNAi | 12.25 ± 0.26 | 11/135 | **<0.05^a^** |

a vs same strain
