# Supplementary material for: The transcription factor SKN-1 drives lysosomal enlargement during aging to maintain function
Source: PLoS Biol. 2025 Dec 5;23(12):e3003540. doi: 10.1371/journal.pbio.3003540 (PMC12694847; doi:10.1371/journal.pbio.3003540)
Supplement: Attachment — Submitted filename: Response to reviewers.docx [file pbio.3003540.s012.docx]

We sincerely appreciate the reviewers’ insightful suggestions. In response to your comments, we have performed substantial additional experiments and included comprehensive experimental details in the revised manuscript. We believe the manuscript has been significantly improved. We are truly grateful to your valuable support.

*Rev. 1:*

*Lysosomes are crucial digestive organelles that preserve cellular homeostasis and functional decline of lysosomes has been associated with aging. Thus, understanding the mechanisms that might lead to their functional decline with age is a relevant topic. One age-dependent lysosomal change that has been documented in several recent studies is morphological changes, such as lysosome tubulation. In this study, Wang et al. describe another morphological change in aging lysosomes: enlargement of their vesicular morphology. The authors propose that this increase in lysosomal size results from lysosomal dysfunction and is a coping mechanism to prevent further lysosomal functional decline. They provide evidence that blocking the enlargement of dysfunctional lysosomes exacerbates their impairment further. Mechanistically, the authors show that the age-related enlargement of lysosomes is regulated by the longevity transcription factor, SKN-1, which limits lysosomal fission in response*

*to lysosomal dysfunction to maintain lysosomal function. Overall, this paper presents interesting findings related to lysosome morphology changes that occur during the natural aging process and highlights the importance of lysosomal morphology adaptation to different stressors. However, there are some points that must be addressed to strengthen the quality of the work, rigor, and the relevance to the field.*

*Major points:*

*1. In general, there is not nearly enough description of the authors' methods used to obtain the data portrayed in the paper. As it is written, these results would not be able to be reproduced by any lab. First, they need to add more information about the various drugs used (concentration, solvent used for dilution, how much was spotted on each plate, when and for how long were the worms exposed). Second, they need to indicate the ages for all experiments in the figures and add more description about the tissues that were imaged in each experiment. Many of the panels describe the fluorescent marker, but not the promoter used. This information should be in the figure or legend. Lastly, they need more description of some of the analysis methods used. For example, in Figure 4I, they analyze the percentage of worms exhibiting fission, but do not describe whether they were looking at the whole worm or just a certain area. Additionally, throughout the paper, the number of*

*lysosomes evaluated is reported but the number of worms where those numbers come from is not clear. This information is key for reproducibility and robustness of the findings.*

**Response:** We sincerely apologize for the insufficient experimental details provided in our original submission. Following your suggestions, we have made the following additions to the revised manuscript:

(1) Added a "Chemical treatments" section in the Methods, which elaborates on the conditions for various compound treatments;

(2) Provided information regarding the age of animals and the tissues observed in the Figure Legends, and indicated the promoters of the reporters used in the Figures;

(3) Expanded the "Examination of lysosomal phenotypes" section in Methods to provide a detailed explanation of the analytical approaches for various lysosomal phenotypes—including the size of regions selected for analysis and the number of animals used for each assay.

*2. Figure 1B shows that lysosomes also have a tubular morphology at day 5 and 9 of adulthood, but the authors do not address any of the tubular morphology in the rest of the paper. Additionally, the rest of the paper only shows vesicular lysosomes in experiments that were conducted at days 5 and 9, meaning these images were purposefully selected away from tubular lysosomes. I think it is misleading that the authors would selectively analyze only worms with vesicular lysosomes and ignore the other major morphological change that they describe in figure 1B. The authors need to examine and quantify tubular lysosomes throughout the rest of their lysosomal imaging experiments, but especially with skn-1 KD, skn-1 gof, and sam-4 KD. It would be informative to show if any of these conditions change proportion of vesicular and tubular lysosomes at various ages. These changes would give more specificity to how lysosomes change in age with skn-1 KD, skn-1 gof, and sam-4 KD.*

**Response:** This is an important suggestion. Following your suggestion, we analyzed changes in the proportion of different lysosomal morphologies in aged *C. elegans* under *skn-1* RNAi, *skn-1* gof, and *sam-4* RNAi treatments. It was found that none of these treatments affected the proportion of tubular lysosomes and vesicular lysosomes (**new Figures S3G, S3J, and S4C**). These results indicate that SKN-1 and SAM-4 specifically influence the size of vesicular lysosomes but have no effect on tubular lysosomes during aging.

*3. On a related point, the authors only quantify vesicle size, but do not quantify vesicle number. In some of the representative images, it appears that as lysosomes enlarge, there may be fewer total vesicles (in skn-1 gof for example). Although it could be because they are morphing into tubular structures as well (it is hard to tell from some of the small images shown). The authors should quantify vesicle numbers to determine if there is a potential effect on lysosome biogenesis as well as quantify lysosome tubulation to appreciate the full impact on lysosomes.*

**Response:** We thank the reviewer for this valuable suggestion. Our findings indicate that the enlargement of vesicular lysosomes is accompanied by a reduction in fission events, suggesting that restricted fission is likely responsible for their increased size. A concomitant consequence of fission limitation is a decrease in the number of vesicular lysosomes (**Figure 4A**). Accordingly, we observed decreased vesicular lysosome numbers in ROS-treated C. elegans, which was reversed by skn-1 RNAi (**new Figure S4M**) and sam-4 RNAi (**new Figure S4N**). Similarly, skn-1 gof animals also exhibited reduced lysosome numbers (**Figure S4O**). These results collectively demonstrate that the enlarged vesicular lysosomes are accompanied by fewer numbers, likely due to limited fission.

Regarding tubular lysosomes, we note that lysosomes do not undergo tubulation upon ROS treatment. Additionally, quantification of tubular lysosome numbers in aged worms is challenging due to their tendency to form network-like structures. However, as you suggested in point #2, our semi-quantitative assessment revealed that neither skn-1 RNAi nor sam-4 RNAi altered the proportion of tubular lysosomes in aged worms (**new Figures S3G and S4C**), indicating that SKN-1 and SAM-4 specifically affect vesicular lysosomes.

Therefore, based on these findings, we propose that SKN-1 and SAM-4 promote the enlargement of vesicular lysosomes by restricting their fission.

*4. The authors only measure lysosome functionality through GFP::LGG-1 cleavage and lysosomal acid phosphate activity. However, because these biochemical assays are done with tissue extracts, it does not inform much about the digestive capacity of the enlarged lysosomes specifically. The authors should directly quantify the effect on cargo turnover when enlarged lysosomes are blocked (skn-1 KD) or enhanced (skn-1 gof mutant). This could be done by using a tandemly tagged autophagy reporter strain (for example, mCherry::GFP::SQST-1) and quantifying GFP/mCherry ratio.*

**Response:** We thank the reviewer for this suggestion. To directly assess lysosomal activity, we employed C. elegans expressing the mCHERRY::GFP::LGG-1 autophagy reporter. LGG-1 is an AP protein and GFP fluorescence is quenched in acidic environment after AP fusion with lysosomes, thus a decrease of the GFP/mCHERRY ratio indicates enhanced autophagic flux efficiency.

Using this reporter, we found that **ROS treatment impaired lysosomal degradation capacity across tissues** (hypodermis, intestine, and muscle), which **was further exacerbated by** skn-1 **RNAi and *sam-4* RNAi (new Figures 5I, S6A, and S6B).** Similarly, aging impaired lysosomal degradation in various tissues, which was further compromised by *skn-1* RNAi (**new Figures 5J, S6C, and S6D**). Conversely, skn-1 activation or ppk-3 RNAi, which promotes lysosomal enlargement in the absence of lysosomal dysfunction, **enhanced lysosomal degradation capacity in various tissues (new Figures 5K, 5L, and S6E-S6H)**. These results **corroborated Western blot analyses of GFP::LGG-1 cleavage,** thereby **confirming the alterations in lysosomal activity reported in the manuscript.**

*5. As an additional point, in the LGG-1 cleavage assays, the uncleaved and cleaved products should be shown in the same blot rather than cropping them out individually so that we can see the cleavage product relative to the uncleaved product in the same gel (similar to what has been done in other publications: PMID: 30929899).*

**Response: We thank the reviewer for this suggestion and have modified the figures to show the total GFP::LGG-1 and cleaved GFP on the same blot.**

*6. The authors show that inducing early enlargement of vesicular lysosomes with ppk-3 RNAi increase activity and that skn-1 KD inhibits late-age lysosomal enlargement but never show that skn-1 KD reduces lysosomal activity in late-age worms. This is an essential experiment.*

**Response:** Using the mCHERRY::GFP::LGG-1 strain, we demonstrated that aging led to impaired lysosomal degradation capacity in various tissues, which was further exacerbated by *skn-1* RNAi (**new Figures 5J, S6C, and S6D**).

*7. The authors show that ROS exposure (TBHP, hydrogen peroxide) and lysosomal membrane damage (LLOME) induce lysosomal enlargement. What about other cellular stresses, such as mitochondrial uncoupling (FCCP), ER stress (tunicamycin), and proteotoxic stress (MG132)? Would these other stressors induce skn-1 activation? These experiments would clarify whether dysfunction of other organelles induces a change in lysosome size.*

**Response:** This is a particularly intriguing hypothesis. Indeed, following our observation that lysosomal enlargement correlates with enhanced lysosomal function, we further investigated whether stresses affecting other organelles could also induce lysosomal enlargement, potentially indicative of inter-organelle communication. W**e found that disruption of the IRE-1/XBP-1 branch of ER UPR induces lysosomal enlargement and functional enhancement (as shown in the figure below), suggesting an adaptive response to ER UPR dysfunction.** We look forward to sharing these findings with the scientific community in the near future.

(A) Effect of *xbp-1(zc12)* mutation on lysosomal morphology and size in the hypodermis of day 1 adults. (B) Effect of *xbp-1(zc12)* mutation on GFP::LGG-1 cleavage in day 1 adults.

*8. This reviewer suggests validating the effect on lysosome enlargement with the skn-1 mutant strains available from CGC to control for possible skn-1 RNAi off-target effects.*

**Response:** As suggested, we found that the *skn-1* mutation abolished lysosomal enlargement in response to ROS treatment (**new Figures S3A and S3B**) or during aging (**new Figures S3E and S3F**), confirming the results from *skn-1* RNAi.

*9. In Figure 3A, the authors describe their screening strategy to identify transcription factor(s) that affect lysosomal enlargement, but this strategy is unclear. More description is needed about whether transcription factor RNAi was also used during the 6 hours that day 1 adults were exposed to TBHP. It would also be good to mention the total number of TFs that were screened.*

**Response:** Thank you for the suggestion. We have added the information regarding the 6-hour TBHP treatment in day 1 adults to the Figure 3A and specified the screening of 757 transcription factors in the main text.

*10. The discussion could be significantly improved for clarity. For example, the last paragraph of the discussion section is very convoluted, and it is not clear what points are trying to be conveyed. The authors could also expand the discussion to discuss the levels of SKN-1 during aging and relate that to their data, speculate on the composition of enlarge lysosomes, and why lysosomes might enlarge instead of maintain the tubular network in late-age.*

**Response:** We thank the reviewer for this valuable suggestion. Accordingly, we have made three major revisions to the Discussion section:

(1) In the second paragraph of the Discussion, we discuss the shift in lysosomal morphology from a predominant tubular form to an increasingly enlarged state during aging. We propose that this transition reflects a dynamic adaptation to different stages of aging: tubulation addresses the increasing demand for cellular waste clearance in early aging, whereas enlargement cope with the progressive functional decline of lysosomes in later stages. Furthermore, we correlate the observed lysosomal enlargement in late aging with previously reported elevation of SKN-1 activity, thereby strengthening our model.

(2) In the fourth paragraph of the Discussion, we explore the possibility that enlarged lysosomes may exhibit altered membrane composition, noting that changes in membrane lipids play a fundamental role in regulating lysosomal fission. We also highlighted the role of SKN-1 in lipid metabolism, proposing a mechanism through which SKN-1 modulates lysosomal morphology via lipid metabolism.

(3) The final paragraph of the Discussion has been revised to more clearly discuss our idea that lysosomal enlargement resulting from restricted fission serves as an adaptive response to acute dysfunctional, whereas prolonged suppression of fission impairs lysosomal regeneration and may be detrimental. We further suggest that for lysosomal enlargement to be beneficial over the long term, compensatory mechanisms must operate to maintain lysosomal quantity. Like in *glp-1* mutants, lysosomal enlargement is associated with activation of TFEB/HLH-30, which promotes lysosomal biogenesis.

*Minor points:*

*1. In the methods, the authors say they quantified gst-4p::GFP intensity using Image J software, but they need to add which features of the software they specifically used. They also need to include specific image settings that were used.*

**Response:** We have incorporated this experimental detail into the Methods section under "Fluorescent microscopy".

*2. While describing lysosome fluorescent images in the results section, the authors need to indicate when those images were taken (age of adulthood). This also needs to be added to each figure caption. Similarly, in the methods section "Examination of lysosomal phenotype," the authors say they quantified lysosomal size using Leica LAS X software, but do not say which features they used in this software.*

**Response:** The age of animals has been added to the Figure Legends, and the procedure for analyzing lysosomal size using Leica LAS X software has been included in the “Fluorescent microscopy” section of the Methods.

*3. In line 57-58 of the introduction, the authors say, "lysosome enlargement is an organelle-autonomous response to their own dysfunction." They did not specifically prove this because they did not test whether other organelle/cellular stresses induced lysosome enlargement and/or skn-1 activation, so the wording of this sentence needs to be changed unless further experimentation is done.*

**Response:** We agree with the reviewer and have deleted the term “organelle-autonomous” from the revised Introduction.

*4. This reviewer suggests migrating Figure S1B into main figure 1. Likewise, I would recommend moving the skn-1 gof data in Figure S4A-C into main figure 5 as this is an important observation that complements the skn-1 RNAi results.*

**Response:** We agree with your suggestion and have adjusted the Figures accordingly.

*5. The order of samples on the western blots sometimes does not match the order of the graphs (for example, 6H-I), which makes it confusing to interpret. I would recommend changing the order of the bar graphs to match the order of samples on the blots to make the presentation more straightforward.*

**Response:** We apologize for this oversight and have revised the relevant figures.

*Rev. 2:*

*The manuscript describes the enlargement of vesicular lysosomes during aging and in mutant long lived C. elegans nematodes. The authors use fluorescent microscopy techniques and RNAi to first characterize lysosomal enlargement during aging. They find that enlargement is an active response to lysosome disruption. This enlargement is mediated by snk-1 and if enlarging is inhibited lysosomal function is further impaired. Specifically long-lived glp-1 animals, but not other worms that have an extended lifespan, show enlarged lysosomes and this is important for the glp-1 mutation induced lifespan extension.*

*The data presented is interesting however it would be beneficial to consider the contribution of other tissues to the observed phenotypes. Additionally, the methods section lacks key details that are important for a clear understanding of the data by the reader.*

*1) One limitation of the study is the focus on a specific subset of lysosomes in the hypodermis. While this provides valuable insights, it would be beneficial to consider the potential contributions of lysosomes from other tissues. The study reports that a subset of hypodermal lysosome enlarges with age and the authors correlate size with lysosome function in whole worms. Several perturbations and measurements (RNAi, ROS exposures, western blots etc) are performed in whole worms and not in a tissue specific way. Thus, it is possible, and likely, that lysosomes in other tissues contribute to the observations.*

*For example, it seems that NUC-1::mCherry is expressed in other tissues as well (as per wormbase and single cell data). Thus, it is possible that the cleavage of the reporter is also caused by lysosomes other than the quantified hypodermal ones (Figure S1). Can the authors exclude the possibility that lysosomes from muscles or the intestine contribute to the signal? These might have a different response in terms of size.*

**Response:** We sincerely appreciate the reviewer's insightful suggestions. In response to this concern, **we first examined lysosomal morphological changes in other tissues, including the intestine and muscle, during aging. Our results showed that lysosomes in these tissues also exhibited enlargement in aged animals (new Figures S1A-S1C), consistent with the phenotype observed in hypodermal lysosomes (Figure 1).** These findings suggest that lysosomes across multiple tissues exhibit a similar morphological response to aging similarly.

**Regarding the NUC-1 cleavage assay, we agree with the reviewer that this method does not specifically indicate hypodermal lysosome activity.** To better evaluate the relationship between lysosomal dysfunction and enlargement at the tissue level, we employed the mCHERRY::GFP::LGG-1 strain to monitor autophagic cargo turnover as an indicator of lysosomal degradative capacity. Since LGG-1 is an autophagy protein and GFP fluorescence is quenched in an acidic environment after autophagosome-lysosome fusion, the GFP/mCHERRY ratio inversely correlates with autophagic flux efficiency**.** We found that ROS treatment impaired lysosomal degradation activity **in various tissues, including the hypodermis (new Figures S2D), intestine (new Figures S2E), and muscle (new Figures S2F). These functional defects were associated with lysosomal enlargement in these tissues (Figures 2E-2F and new Figures S2G-S2J). Together, these findings** demonstrate that ROS-induced lysosomal dysfunction is associated with morphological enlargement across diverse tissues.

*The same would apply to other interventions such as skn-1 RNAi or pkk-3 RNAi, which might not affect lysosome size in other tissues, but these lysosomes still contribute to the LGG-GFP cleavage signal (Figure 5). To be able to make the conclusion that enlargement is an active adaptive response to preserve lysosomal degradation capacity, lysosomes in other tissues should be quantified, tissue specific interventions should be used, or tissue specific reporters would be necessary.*

*To strengthen their conclusions, hypodermis specific image-based analysis could be performed by using the flux reporter mcherry::GFP::lgg (as in Figure 5) combined with lysotracker to stain for lysosomal acidity. This would allow to disentangle function at a cellular level and with excluding any potential impact of tubular lysosomes.*

**Response:** We thank the reviewer for this suggestion. In response to this concern, we measured the size of degradative autolysosomes (ALs) and lysosomal activity at the tissue level by employing the mCHERRY::GFP::LGG-1 strain. Our results demonstrate that ALs were enlarged upon ROS exposure in the hypodermis, intestine, and muscle. These effects were reversed by *skn-1* RNAi and *sam-4* RNAi (**new Figures 5A, S5A, and S5B**). Similarly, aging increased AL size across various tissues in a SKN-1-dependent manner (**new Figures 5B, S5C, and S5D**). Additionally, both *skn-1* activation and *ppk-3* knockdown resulted in enlarged ALs in these tissues in the absence lysosomal dysfunction (**new Figures 5C, 5D, and S5E-S5H**). These results suggest that (1) degradative lysosomes enlarge in various tissues in response to dysfunction, and (2) genes such as *skn-1* and *ppk-3* modulate lysosomal size in multiple tissues.

To further correlate lysosomal enlargement with function at the tissue level and validate the GFP::LGG-1 cleavage results, we examined autophagic cargo turnover using the mCHERRY::GFP::LGG-1 strain. Our results showed that ROS treatment impaired lysosomal degradation in the hypodermis (**new Figures 5I**), intestine (**new Figure S6A**), and muscle (**new Figure S6B**). **These impairments were further exacerbated by** skn-1 **RNAi and** sam-4 **RNAi (new Figures 5I, S6A, and S6B).** Aging also impaired lysosomal degradation capacity in various tissues, which was further reduced by *skn-1* RNAi (**new Figures 5J, S6C, and S6D**). Conversely, both skn-1 activation (**new Figures 5K, S6E, and S6F**) and ppk-3 knockdown (**new Figures 5L, S6G, and S6H**), which promote lysosomal enlargement in the absence of lysosomal dysfunction, enhanced lysosomal degradation in these tissues. **These cellular-level observations corroborate the conclusions from GFP::LGG-1 cleavage assays, confirming that lysosomal enlargement enhances degradative capacity.**

*2) The methods section should be expanded to provide more details, ensuring that readers can fully understand the approach.*

*Lysosomal quantification: How is the area of lysosomes measured (manually, automatically, where in the worm e.g. head hypodermis, what is the depth imaged)? Are the tubular lysosomes excluded in the analysis and if so how (manually, were the investigators blinded)? How many worms are imaged per quantification? What happens to animals that show tubular structures (e.g. also in vha-7 RNAi) or animals that have both tubular and vesicular structures? Were those animals excluded from analysis? Are experiments merged and in case were there any batch effects?*

**Response:** We sincerely apologize for the insufficient experimental details provided in our original submission. We have incorporated all experimental details and parameters related to lysosomal phenotypic analysis into the “Fluorescent microscopy” and “Examination of lysosomal phenotypes” sections of the Methods.

It is important to note that young wild-type worms contain almost no tubular lysosomes, and none of the other treatments included in this study, apart from aging, induce the formation of tubular lysosomes. Therefore, to avoid potential misinterpretation due to the occasional presence of tubular structures in previously selected representative images (such as in the case of vha-7 RNAi), we have replaced the image with a more representative one showing only vesicular lysosomes. For the analysis of vesicular lysosome size in aged worms, we selected animals predominantly exhibiting vesicular lysosomes and manually outlined these structures for size measurement. This procedural detail has also been added to the “Fluorescent microscopy” section of the Methods.

Additionally, the analytical process for different lysosomal phenotypic data—specifically, whether the data were derived from a representative experiment or pooled from multiple replicates—has been detailed in the “Examination of lysosomal phenotypes” section of the Methods.

*The authors use levamisole to fix the worms, which is known to cause LGG-1 puncta 5 minutes after mounting (PMID 25569839). At least some central experiments should be repeated using NaN3 immobilization to test if this shows similar phenotypes regarding their findings.*

**Response:** This is an important concern. In response, we performed additional experiments using NaN₃ to validate that both ROS treatment and aging induce lysosomal enlargement in a manner dependent on SKN-1 and SAM-4 (as shown in the figures below).

(A-B) Effects of *skn-1* (A) and *sam-4* (B) RNAi on lysosomal morphology and size in response to six-hour TBHP treatment in the hypodermis of day 1 adults. Left: representative images. Right: quantification data. (C-D) Effects of *skn-1* (C) and *sam-4* (D) RNAi on vesicular lysosomal morphology and size in the hypodermis during aging. Left: representative images. Right: quantification data.

Regarding lysosomal function, our original GFP::LGG-1 cleavage assays did not involve anesthetic treatment, thereby ensuring the reliability of these results. Furthermore, new functional data from the mCHERRY::GFP::LGG-1 strain (**new Figures 5I-5L, S6**), which is relevant to the LGG-1::GFP puncta the reviewer mentioned, were performed under NaN₃ treatment. This information was clearly indicated in the Methods. Importantly, the conclusions from these new experiments are consistent with those from the GFP::LGG-1 cleavage assays (Please see our response to your concern #1). Therefore, our findings are not affected by the method of worm immobilization.

*Tissue: The hypodermis is mentioned once in the context of Figure 1 and in context of the strain sam-4::GFP strain generation. It seems that the authors use the hypodermis for all quantifications. Maybe I missed this, but it would be helpful to have this information mentioned specifically in the methods but potentially also in the figure/legends.*

**Response:** We have added the tissue information to the Figures and Legends.

*Statistics: What is the criteria for showing a p-value versus not showing it? Could the authors please make it clear in the figure which conditions are compared separating the lines. Which pairwise comparison test is used within the ANOVA? Please elaborate on why the statistical tests switch between t-test, ANOVA and chi-square. Were experimenters blinded? Which test was used for the lifespan statistics (in the figure legend it states Mantel-Cox but in the table Bonferroni corrected is mentioned).*

**Response:** We have made the following revisions regarding the statistical methods:

(1) Separated the lines in the figures to clearly indicate which groups were compared;

(2) Added criteria for selecting different statistical methods in the Statistics section of the Methods;

(3) Included the specific post hoc tests used for each statistical analysis in the figure legends;

(4) Specified in the Statistics section that the researchers were unblinded during the analysis;

(5) Noted in the figure legends that the lifespan data were analyzed using the Mantel–Cox test, with Bonferroni post hoc tests applied for p-value calculation.

*3) Some findings are based on sam-4 function as a myrlysine ortholog. The authors mention that further analysis is needed to confirm that sam-4 is located on lysosomal membranes. In addition to the missing GFP signal in the hypodermis, single cell RNAseq data shows only limited transcripts of sam-4 in the hypodermis and sam-4 RNAi has no effect on lysosomal size in unperturbed conditions. Can the authors rule out the possibility that the effect on hypodermal lysosomes or lifespan is an indirect consequence of SAM-4 activity in other tissues, such as impacting neuronal functions, or potentially other functions?*

**Response:** We appreciate this important suggestion. Using **tissue-specific RNAi strains**, we investigated the impact of sam-4 knockdown on lysosomal size in the hypodermis and on C. elegans lifespan. **Our results revealed that only** sam-4 RNAi **specifically in the hypodermis suppressed the enlargement of hypodermal lysosomes in response to lysosomal dysfunction (new Figures S4D-S4I),** indicating that **SAM-4 regulates lysosomal size in a tissue-autonomous manner.**

**For lifespan analysis**, we found that **RNAi-mediated depletion of** sam-4 **in all tissues examined (hypodermis, intestine, and muscle) modestly reduced lifespan (new Figures S7C-S7E).** This observation is **consistent with the finding that lysosomes enlarge in all these tissues during aging (Figure 1 and new Figure S1).** Collectively, these data **support the model that lysosomal enlargement in multiple tissues contributes to organismal lifespan regulation.**
