# Supplementary material for: The transcription factor SKN-1 drives lysosomal enlargement during aging to maintain function
Source: PLoS Biol. 2025 Dec 5;23(12):e3003540. doi: 10.1371/journal.pbio.3003540 (PMC12694847; doi:10.1371/journal.pbio.3003540)
Supplement: Attachment — Submitted filename: Response_to_reviewers_auresp_3.docx [file pbio.3003540.s013.docx]

***Rev. 1:***

*The authors have performed critical additional experiments that significantly improve the robustness of the work. Specifically, they have performed a much more rigorous analysis of all lysosome morphology and have examined autophagic turnover specifically at enlarged lysosomes and added essential details to their methodology. The addition of these critical experiments provides improved clarity on the functionality of the enlarged lysosomes. They have also significantly improved the discussion and presented a reasonable model. Overall, they have satisfactorily addressed all major concerns, improved the quality of the written manuscript, and I think it is now suitable for publication.*

**Response:** Thank you for your positive feedback on our manuscript.

***Rev. 2:***

*The authors have addressed my comments by including new experiments and additional information to the manuscript. I appreciate the authors' effort in including different tissues to test their contribution. The work showing that a subset of lysosomes enlarges with age as a compensatory mechanism is exciting, and I would recommend publication. However, in the revised version some minor comments have emerged which could be addressed with text edits.*

**Response:** Thank you for your positive feedback on our revisions.

*(1) With the added information in the Methods section, it is now clear that data from multiple experiments were pooled. For some experiments (e.g. S5K, L and S6) it seems that each replicate has only few worms (4 individuals per replicate). This number seems low in comparison to other experiments especially since a small region (10x10µm) is analyzed. Could the authors please highlight the separate biological replicates in their data table (potentially also the graphs) to ensure that individual biological replicates are not skewing the results.*

**Response:** We appreciate the comment regarding the sample size. We agree that a sample size of four worms per group per replicate was limited for the autophagic turnover assay using the mCHERRY::GFP::LGG-1 strain. In response, we have increased the number of animals analyzed to seven per group per replicate (21 in total for the pooled data). Additionally, as suggested, we have provided not only the pooled data but also the data from each independent replicate in S1 Data, which confirms that the findings are consistent across all replicates.

*(2) The authors now clarified that they exclude the animals that show predominantly tubular structures from the image analysis. However, in the worm lysates western blots and the phosphatase assays, tubular lysosomes might contribute to the observations. Could the authors include a couple of sentences to acknowledge this limitation?*

**Response:** This is a great suggestion. We have added the following sentence after the phosphatase activity results (Line 119): "It should also be noted that the acid phosphatase assay measured the enzyme activity from the whole animal, reflecting the combined activity of both vesicular and tubular lysosomes." Additionally, we have included the following clarification after the GFP::LGG-1 western blot data (Line 361): "Given that GFP::LGG-1 cleavage indicates lysosomal degradation capacity at the organismal level, and it cannot discriminate between the specific contributions of vesicular versus tubular lysosomes during aging, we further assessed the functional consequence of vesicular lysosomal enlargement at a tissue level."

*(3) The authors state in line 421 that a 100x100 µm area per worm was analyzed. Given that the worm is approximately 68µm wide this seems inconsistent. This might be a typo as later a 10x10 µm area is mentioned. Please clarify or correct this point.*

**Response:** We apologize for this oversight. Fission events were analyzed in a 50 × 50 μm area, and this has now been corrected in the manuscript (Line 554).

*(4) The acid phosphatase Kit P0326 that has now been described in the method section analyzes the activity of all acid phosphatases. What about acid phosphatases that are not in the lysosomes but e.g. secreted? The contribution of these might be low but it should be acknowledged. It should be considered to rephrase the statement made in the line 104 and 110-112 to highlight that other phosphatases outside of lysosomes might contribute to this result as well. In addition, it would be helpful to add how samples were normalized (by protein content?)*

**Response:** Thank you for the suggestion. Accordingly, we have revised the sentence (Line 110-113) to the following: " Aging is associated with a decline in lysosomal function in *C. elegans*, which we confirmed by assessing the activity of acid phosphate (Fig 2A), a predominantly lysosomal enzyme, while acknowledging the potential contribution from non-lysosomal sources."

Additionally, we have clarified in the Methods section that the enzyme activity was normalized to the protein content (Line 559).
